# Disparities in COVID-19 related health literacy, knowledge, and the assessment of the measures taken in Germany: a cross-sectional study

**DOI:** 10.1007/s10389-023-01827-2

**Published:** 2023-01-21

**Authors:** Lisa Schmidt, Nina-Alexandra Götz, Niels Hannemann, Birgit Babitsch

**Affiliations:** 1 Department of New Public Health, Osnabrück University, Nelson-Mandela-Straße 13, D-49076 Osnabrück, Germany; 2 Osnabrück University of Applied Sciences, Albert-Einstein-Str. 1, D-49076 Osnabrück, Germany

**Keywords:** Health literacy, SARS-CoV-2, COVID-19, Social inequality

## Abstract

**Aim:**

Health literacy is necessary to access, understand, assess, and apply information on COVID-19. Studies have shown that health literacy is unequally distributed across social groups. This study aimed to analyze the differences in COVID-19-related health literacy (hereinafter referred to as “COV-19-HL”), knowledge about COVID-19, and the assessment of the measures taken regarding the sociodemographic characteristics as well as the influence of COV-19-HL on knowledge and assessments.

**Subject and methods:**

The study used the data obtained from the cross-sectional online survey “Digital divide in relation to health literacy during the COVID-19 pandemic.” The data covers 1570 participants aged ≥18 years in Germany between April 29, 2020 and May 8, 2020. To analyze the differences by way of sociodemographic variables, t-tests and analyses of variance were carried out. Multivariate logistic regression models were used to determine the effect of COV-19-HL on knowledge and the assessment of measures.

**Results:**

The overall COV-19-HL was high with an average value of 37.4 (with 50 representing the highest COV-19-HL). COV-19-HL and knowledge about COVID-19 were slightly lower in men, migrants, people with low subjective social status, and with low education. Government requirements and recommendations were rated as more effective by women, older people, and individuals with a chronic illness. The chance of better knowledge about COVID-19 and rating measures as effective increased with higher COV-19-HL.

**Conclusion:**

The findings of this study show that COV-19-HL and knowledge about the virus are unequally distributed in Germany. Health communication should strengthen pandemic-related health literacy that is tailored to specific target groups.

## Introduction

The COVID-19 pandemic has posed new challenges for society and health care. During the first wave of the pandemic (March to April 2020), a broad range of public health measures were implemented to contain COVID-19. In Germany, those measures included restrictions in public life (closing of restaurants, cinemas, etc., as well as schools and kindergartens) and social distancing (reducing contact and keeping a distance of 1.5 m to other people) (Presse- und Informationsamt der Bundesregierung 2020a, b). On April 30, 2020, the opening of playgrounds, museums, zoos, and churches was announced and, at the same time, the mandatory mask usage while in shops as well as on buses and trains was introduced in all of the German federal states (Mitteldeutscher Rundfunk 2020). Additional recommendations of the government have included thorough hand washing and keeping a physical distance of 1.5 m away from each other. The routine disinfection of hands and surfaces is not determined to be necessary to inactivate COVID-19 if proper cleaning is possible, with the exception of the health care setting and in case of being infected with the virus (Robert Koch-Institut 2020a, b).

Recommendations for dealing with the pandemic as well as political decisions were and are also being discussed in detail in the media. There are a multitude of different information flows into the population via traditional and digital media that they have to deal with. Internationally, therefore, there has already been talk of an “infodemic” or “information overload” (Eysenbach 2020; Rathore and Farooq 2020; Zarocostas 2020). At the beginning of March 2020, 67% of those surveyed for repeated monitoring described the COVID-19 situation as exaggerated by the media; in May 2020, it was 48% (Universität Erfurt et al. 2021). However, in addition to useful information on how to deal with COVID-19, such as social distancing, etc., there is also information being shared that has not been scientifically confirmed or is simply false (Rathore and Farooq 2020). This can lead to uncertainty and the disregard of the COVID-19 rules among the population, for example, due to the spread of misinformation about medical mouth–nose protection leading to carbon dioxide poisoning (WHO 2020).

In addition to education on scientifically validated information, people’s health literacy is an important factor for the safe handling of this infodemic. Health literacy is described as a personal resource for handling health information (Abel and Sommerhalder 2015). It includes the knowledge, motivation, and skills to acquire, understand, evaluate, and apply health information so that health-related decisions can be made (Sørensen et al. 2012). It is also a subject of organizational and community structures as well as public policies (Sentell et al. 2020). The World Health Organization (WHO) has been describing health literacy as one of the most important determinants of health for several years (WHO 2017). During the COVID-19 pandemic, health literacy has gained in importance. Nutbeam (2000) distinguishes three types of health literacy: basic/functional literacy, communicative/interactive literacy, and critical literacy. Basic/functional literacy includes reading and writing skills that are necessary in everyday life to understand health-related information. The communicative/interactive literacy includes social and cognitive skills for active participation in life. These two types of health literacy are required for searching for and sharing information. The third type, critical literacy, describes cognitive and social skills for critically dealing with information in order to be able to use it optimally for one’s own life. Critical literacy is particularly important to manage the information overload experienced during the COVID-19 pandemic. Health literacy can help you to understand the motives behind political measures and to internalize the importance of social responsibility in one’s own behavior (Paakkari and Okan 2020). It can also help to better assess health risks and policies. Failure to comply with the rules of conduct can result from the adoption of incorrect information from social media and from uncertainty about the applicable rules of conduct (Ölcer et al. 2020). People’s COVID-19-related health literacy (hereinafter referred to as “COV-19-HL”) could, therefore, be the key to mitigating the spread of the virus (Košir and Sørensen 2020).

However, looking at the HLS-GER study on the health literacy of the German population with data from 2014 and 2020, it becomes apparent that the number of people with limited health literacy increased from 54% in 2014 to 64% in 2020 (Schaeffer et al. 2016; Hurrelmann et al. 2020). The study also drew attention to the fact that health literacy is unequally distributed among the population. Therefore, older age, chronic diseases, low subjective social status, low education, and migration background were found to be factors associated with limited health literacy. The HLS study also made it evident that people with chronic diseases had poorer health literacy than those without chronic diseases (Schaeffer et al. 2016; Hurrelmann et al. 2020). Therefore, the authors conclude that those with limited health literacy exhibit unhealthier behaviors and have poorer health status (ibid.).

Research about health literacy in the context of the COVID-19 pandemic is rare. Using cross-sectional data from April 2020, Okan et al. (2020a) described that COV-19 HL in the German population was adequate in 50% of the online respondents. Problematic or inadequate COV-19-HL was attributed to 15% and 35% of respondents, respectively (ibid.). Other studies also suggest that better health literacy during the pandemic is associated with adherence to preventive behaviors (Hermans et al. 2021; McCaffery et al. 2020; Patil et al. 2021) and a lower risk for COVID-19 symptoms (McCaffery et al. 2020; Greer et al. 2021; McCaffery et al. 2020; Nguyen et al. 2020). Looking at the “Integrated model of health literacy” from Sørensen et al. (2012), there are three health domains that are influenced by health literacy; health care, disease prevention, and health promotion. In the context of COVID-19, health literacy generates knowledge and skills concerning the (COVID-19) disease prevention by being able to access, understand, and interpret information on COVID-19 and to make informed decisions. Therefore, health literacy can lead to increased knowledge of risks of COVID-19 and adherence to prescribed actions. Data are lacking on the possible correlations between COV-19-HL with knowledge about COVID-19 and the attitudes toward protective measures as well as on the differences between social groups.

Therefore, the following questions will be examined in this study:

Which differences in COV-19-HL, knowledge about COVID-19, and the assessment of the effectiveness of measures against COVID-19 are existing in relation to the sociodemographic characteristics? How are knowledge about COVID-19 and the assessments of the effectiveness of the measures against COVID-19 influenced by COV-19-HL?

## Methods

### Study design

This analysis is part of the cross-sectional study “Digital divide in relation to health literacy during the COVID-19 pandemic.” Data was collected by using an online questionnaire between April 29, 2020 and May 8, 2020. The exit and contact restrictions declared by the federal government in March were slowly being relaxed during this period. The composition of the quota sample corresponds to the current distribution of age, gender, and residence in a federal state (not crossed) according to Eurostat 2018 database. The participants were recruited from an online panel through an external survey provider (respondi AG). The survey provider sent the invitation to participate to their registered users via email. Participation in this survey was voluntary, and users received a small remuneration by the data collection company for completing questionnaires. The invitation email included information on the duration of the survey and possible remuneration. Individuals between 18 and 74 years of age living in Germany were eligible for inclusion in the study. On the first page of the survey, the participants were asked about demographic information which would be used to decide whether they would be included or excluded from the survey, depending on the quota requirements. In total, 1953 people participated in the online questionnaire; 383 of them were excluded because of full quotas or incomplete data and, therefore, 1570 participants were included in the final sample. All of the questions had to be answered in full. In the sample, the proportion of people with low education is larger than the national average. The survey ensured anonymity and privacy. The external provider ensured compliance with data protection in accordance with the General Data Protection Regulation (DSGVO). Data were gathered and stored anonymized. After the survey period, the provider sent the anonymized data set to the research group.

### Dependent variables

#### COVID-19 related health literacy

**Fig. 1:**
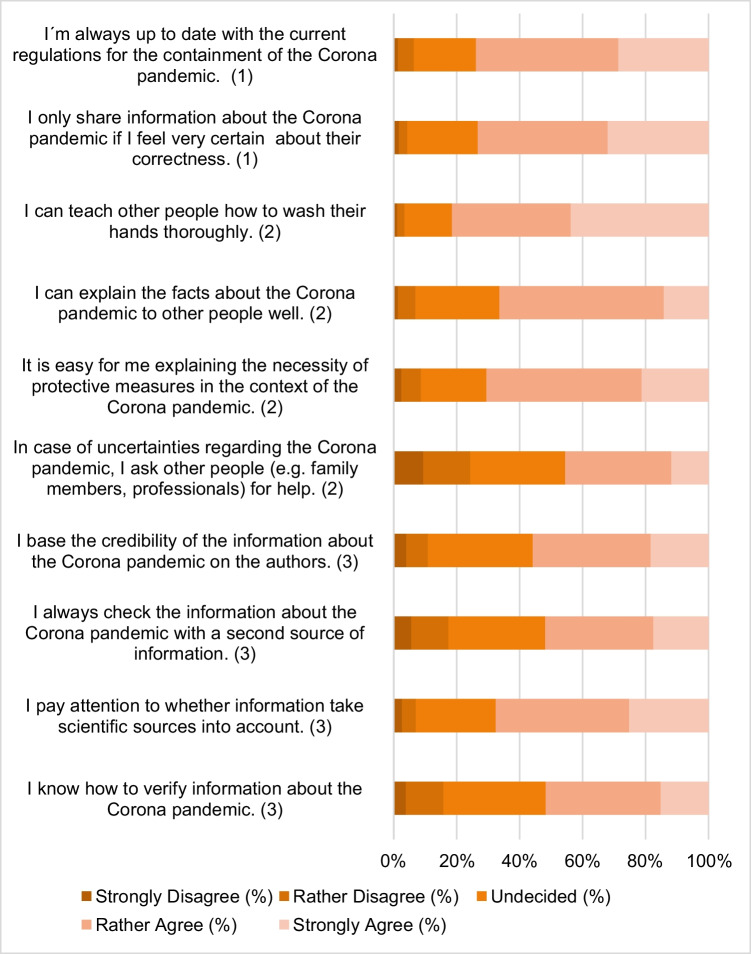
Frequencies for COV-19-HL items (*N* = 1570). Notes: Types of health literacy are in brackets with 1 = basic/functional literacy, 2 = communicative/interactive literacy, and 3= critical literacy

The COV-19-HL was measured on the basis of 10 items (see Fig. 1) in which statements had to be rated on a 5-point scale (does not apply at all (1), does rather not apply (2), neither (3), rather applies (4), fully applies (5)). The statements about one’s COV-19-HL are derived from the recommendation of Okan et al. (2020b). For statements about COV-19-HL, a total score was then calculated from the 10 items, so that 10 to 50 points could be achieved (with 50 points = highest COV-19-HL). The measuring instrument has shown good reliability in this survey (Cronbach’s alpha = 0.830).

#### Knowledge of COVID-19

Knowledge of COVID-19 was tested using five statements to be assessed (Table 4). The topics of the statements were derived from the FAQ section of the website of the German Federal Ministry of Health and the Robert Koch Institute. The statements include knowledge about behaviors to contain the spread of the virus and scientific knowledge on a simple level. One point was awarded for each correct answer and zero points for incorrect answers or statements that could not be assessed. A total score was formed with a value range from 0 to 5, which corresponds to the number of correctly assessed statements.

#### Assessments of the effectiveness of protective measures against COVID-19

**Fig. 2:**
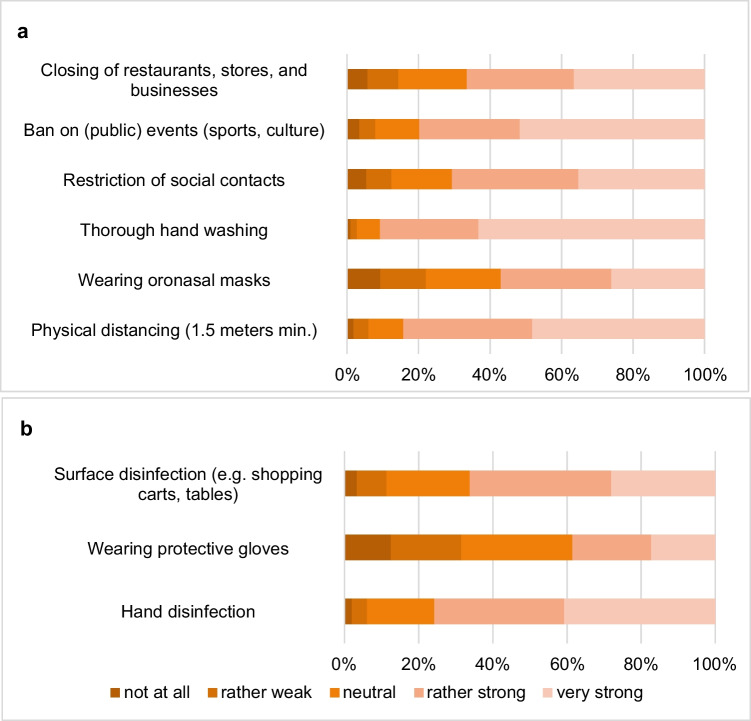
Assessment of the effectiveness of (**a**) government requirements and recommendations and (**b**) additional measures (*N* = 1570)

Nine measures against COVID-19 were assessed regarding effectiveness by the participants (see Fig. 2). We included six measures that were recommended by the Federal Ministry of Health and the Robert Koch Institute and three additional measures that were carried out frequently but not officially recommended. The topics range from personal to organizational protective measures against the spread of the virus. Assessment of effectiveness ranged from 1 = not at all to 5 = very strong on a five-point Likert scale in the developed questionnaire.

### Independent variables

#### General health literacy

General health literacy was based on a score of six items on a five-point Likert scale (with 5= very easy to 1= very difficult). Items derived from the instrument of Schaeffer et al. (see (Schaeffer et al. 2016)). Participants rated their competencies to get, understand, evaluate the content, assess the credibility, and critically question health related information and use it as a basis for decisions. Good reliability was seen with Cronbach’s alpha = 0.895 in this survey.

#### Sociodemographics and health

Age was categorized into four groups in line with the federal health report (Robert Koch Institut 2014): 18–29, 30–44, 45–64, and >65 years. The CASMIN classification (Lechert et al. 2006) was used to measure education and classify it in groups (low, medium, and high education). Subjective social status was assessed using the MacArthur Scale of Subjective Social Status (Adler et al. 2000), which includes self-assessment on a 10-step ladder. The categories were defined according to Hoebel and colleagues (Hoebel et al. 2013): people with a low (scale values 1–4), medium (scale values 5–6), and high (scale values 7–10) subjective social status. The recommendations by Schenk and colleagues (Schenk et al. 2006) were used to ascertain the migration status. Exposure to COVID-19 was surveyed by one question about a known infection of oneself or of someone in the social environment.

#### Statistical analyses

Absolute and relative frequencies for COV-19-HL items, knowledge items, and items for the assessments of the effectiveness of measures as well as the means and standard deviations for the COV-19-HL score and the knowledge score were calculated. To analyze the differences in COV-19-HL, knowledge and the assessments of the effectiveness of measures by sociodemographic variables, t-tests and analyses of variance including the Scheffé post-hoc test were carried out after testing the requirements. The correlation between COV-19-HL and knowledge of COVID-19 was calculated using the Pearson correlation coefficient. After bivariate analyses, multivariate logistic regression models were used to explore the effect of COV-19-HL on knowledge of COVID-19 and on the assessments of the effectiveness of the measures. All of the analyses were conducted using IBM SPSS Statistics, Version 26.

## Results

### Description of the sample

The characteristics of the study participants are shown in Table 1. A total of 1570 people answered the questionnaire on COV-19-HL, including 50% women and 50% men. Most of the participants were between 45 and 64 years old (42.7%); 19.2% of the respondents were aged 18–29 years, 24.6% were aged 30–44, and 13.4% were aged 65 years and older. When asked about subjective social status, 23.6% of the respondents classified themselves in the lower subjective social status group, 41.5% in the medium, and 35% in the higher subjective social status group. Looking at the educational qualifications, it can be seen that around half had a medium level of education, 30.3% had a low level of education, and 18% had a high level of education. A migration status was present in 132 respondents (8.4%). Respondents of our survey frequently reported having a chronic illness (47.2%) and 15.5% were infected with COVID-19 or had contact with an infected person.

**Table 1:** Sample characteristics (*N* = 1570)

Variable	N
Gender	
Female	785 (50%)
Male	785 (50%)
Age	
18–29 years	302 (19.2%)
30–44 years	387 (24.6%)
45–64 years	671 (42.7%)
65 years and above	210 (13.4%)
Migration status	
Yes	132 (8.4%)
No	1438 (91.6%)
Subjective social status	
Low	370 (23.6%)
Medium	651 (41.5%)
High	549 (35%)
Education	
Low	476 (30.3%)
Medium	812 (51.7%)
High	282 (18%)
Chronic illness	
Yes	741 (47.2%)
No	829 (52.8%)
Infection with COVID-19	
Not known	1326 (84.5%)
Known infection of oneself or contacts	244 (15.5%)

### COVID-19 related health literacy (COV-19-HL)

The COV-19-HL of the participants was overall high in this survey. With 50 points representing the highest COV-19-HL, an average value of 37.4 (SD = 6.0) was achieved. Only 2.4% of the respondents achieved less than half of the points. The frequencies of answers in the single items of the COV-19-HL scale are shown in Fig. 1. Checking the information about the pandemic with a second source and asking other people for help in case of ambiguity seemed to be the most problematic competences. Overall items of critical literacy were assessed slightly worse than communicative and basic literacy.

The differences in COV-19-HL were calculated for the sociodemographic characteristics of gender, age, migration status, subjective social status, education, chronic illness, and infection with COVID-19 (see Table 2). There are small differences in the mean scores of COV-19-HL in individuals with a migration status (M = 36.30) compared to those without a migration status (M = 37.38, *p* < 0.05). With an average value of M = 37.97, women have a slightly better COV-19-HL than men (M = 36.59, *p* < 0.01). When looking at the four age groups, there were no significant differences. The differences in COV-19-HL between the subjective social status groups are small, but statistically significant. There is a difference between low subjective social status (M = 36.81) and high subjective social status (M = 38.06) (*p* < 0.01) and between medium subjective social status (M = 36.90) and high subjective social status (*p* < 0.01). A social gradient in COV-19-HL is also evident in the educational groups (low education M = 36.34; medium education M = 37.30; high education M = 38.82; *p* < 0.01 for low vs. high and medium vs. high education). People with chronic illnesses (M = 37.82) had higher levels of COV-19-HL than people without chronic illnesses (M = 36.80, *p* < 0.01).

**Table 2:** Mean COV-19-HL scores by participant characteristics

Variable	COV-19-HL Score	Comparison between groups	
	Mean (SD*)	t-test	ANOVA
		*p*-value	*p*-value
Gender			
Female (*n* = 785)	37.97 (5.82)	<0.01	
Male (*n* = 785)	36.59 (6.18)		
Age			
18-29 years (*n* = 302)	37.10 (5.83)		0.06
30-44 years (*n* = 387)	36.70 (6.48)		
45-64 years (*n* = 671)	37.72 (6.04)		
65 years and above (*n* = 210)	37.24 (5.39)		
Migration status			
No (*n* = 1438)	37.37 (6.03)	<0.05	
Yes (*n* = 132)	36.3 (6.11)		
Subjective social status			
Low (*n* = 370)	36.81 (5.98)		<0.01
Medium (*n* = 651)	36.90 (5.76)		
High (*n* = 549)	38.06 (6.33)		
Education			
Low (*n* = 476)	36.34 (6.13)		<0.01
Medium (*n* = 812)	37.30 (5.95)		
High (*n* = 282)	38.82 (5.83)		
Chronic illness			
No (*n* = 829)	36.80 (6.27)	<0.01	
Yes (*n* = 741)	37.82 (5.72)		
Infection with COVID-19			
Not known (*n* = 1326)	37.01 (6.08)	<0.01	
Known infection of oneself or contacts (*n* = 244)	38.77 (5.58)		

*SD = standard deviation

### Knowledge of COVID-19

Out of five statements on COVID-19, all of the participants answered an average of 3.3 statements correctly. Only 19% of the participants were able to correctly assess all of the statements. The statement about the number of new cases was the most difficult for the participants and was assessed correctly by only 49.6% (see Table 5). The differences by participant characteristics are shown in Table 3. A social gradient was found with regard to educational groups. People with higher education had higher mean knowledge scores than people with lower education. The differences between the subjective social status groups were small and only significant between medium and high subjective social status. We also found differences in knowledge by migration status with people without migration status having higher mean scores than people with migration status. Age did not seem to play a role in knowledge of COVID-19. People with chronic illnesses had higher knowledge than people without chronic illnesses and females achieved higher scores than males.

**Table 3:** Mean COVID-19 knowledge scores by participant characteristics

Variable	COVID-19 knowledge score	Comparison between groups	
	Mean (SD*)	t-test	ANOVA
		*p*-value	*p*-value
Gender			
Female (*n* = 785)	3.39 (1.26)	<0.01	
Male (*n* = 785)	3.22 (1.32)		
Age			
18-29 years (*n* = 302)	3.35 (1.22)		0.07
30-44 years (*n* = 387)	3.35 (1.32)		
45-64 years (*n* = 671)	3.32 (1.29)		
65 years and above (*n* = 210)	3.31 (1.33)		
Migration status			
No (*n* = 1438)	3.35 (1.28)	<0.01	
Yes (*n* = 132)	2.83 (1.35)		
Subjective social status			
Low (*n* = 370)	3.26 (1.26)		<0.05
Medium (*n* = 651)	3.23 (1.31)		
High (*n* = 549)	3.42 (1.28)		
Education			
Low (*n* = 476)	2.99 (1.34)		<0.01
Medium (*n* = 812)	3.34 (1.26)		
High (*n* = 282)	3.76 (1.14)		
Chronic illness			
No (*n* = 829)	3.24 (1.32)	<0.05	
Yes (*n* = 741)	3.37 (1.26)		
Infection with COVID-19			
Not known (*n* = 1326)	3.28 (1.30)	<0.05	
Known infection of oneself or contacts (*n* = 244)	3.46 (1.22)		

*SD = standard deviation

### Assessments of the effectiveness of protective measures against COVID-19

Overall, most of the participants rated the recommended and the additional measures against COVID-19 during the first wave as effective (see Fig. 2). Wearing protective gloves and an oronasal mask were the lowest rated measures. It is notable that 22.1% of the participants assessed the effectiveness of wearing an oronasal mask as rather weak or not at all effective. Older people, women, and individuals with a chronic illness rated the government requirements and recommendations as more effective than the comparison groups. Differences by migrations status, subjective social status, and education were smaller and in almost all items not significant.

### COV-19-HL and knowledge of COVID-19

In four out of the five knowledge questions, those with a correct answer had on average a higher COV-19-HL score than those who did not know the answer (see Table 4). The evident average differences range from 1.2 to 3.6 points in COV-19-HL.

**Table 4:** COV-19-HL by knowledge of COVID-19

Knowledge items	COV-19-HL scores		
	Answer incorrect or did not know	Answer correct	t-test
	Mean (SD)	Mean (SD)	*p*-value
The likelihood of a severe course of COVID-19 disease increases significantly with age.	34.31 (6.05)	37.94 (5.84)	<0.01
By wearing a simple face mask, I am protected against a COVID-19 infection (SARS-CoV-2).	36.39 (6.72)	37.57 (5.78)	<0.01
The number of new cases is sufficient to be able to assess the extent of infections as a whole.	37.09 (6.19)	37.48 (5.88)	0.20
The differences between the reported numbers of people infected with COVID-19 (SARS-CoV-2) can be explained by the use of different data sources.	35.48 (5.99)	38.69 (5.69)	<0.01
It is only if I disinfect my hands after washing them thoroughly that I will be sufficiently protected against a COVID-19 infection (SARS-CoV-2).	36.50 (6.64)	37.67 (5.68)	<0.01

Table 5 shows that COV-19-HL is an important predictor of knowledge about COVID-19. The effect of COV-19-HL appears in the adjusted multivariate regression models of four knowledge questions. Here, the chance of rating the statements correctly increases with higher COV-19-HL scores. Furthermore, there is a low correlation between the knowledge score and COV-19-HL with r = 0.245 (*p* < 0.01).

**Table 5:** Distribution of knowledge items and multivariate regression models exploring knowledge about COVID-19 by COV-19-HL

			Regression models with COV-19-HL as independent variable*		
Knowledge items	Incorrect or did not know n (%)	Correct n (%)	OR	95% confidence interval	*p*
The likelihood of a severe course of COVID-19 disease increases significantly with age.	285 (18.2)	1285 (81.8)	1.106	1.079–1.134	<0.01
By wearing a simple face mask, I am protected against a COVID-19 infection (SARS-CoV-2).	380 (24.2)	1190 (75.8)	1.033	1.011–1.056	<0.01
The number of new cases is sufficient to be able to assess the extent of infections as a whole.	791 (50.4)	779 (49.6)	1.008	0.989–1.027	0.408
The differences between the reported numbers of people infected with COVID-19 (SARS-CoV-2) can be explained by the use of different data sources.	687 (43.8)	883 (56.2)	1.088	1.066–1.111	<0.01
It is only if I disinfect my hands after washing them thoroughly that I will be sufficiently protected against a COVID-19 infection (SARS-CoV-2).	518 (33.0)	1052 (67.0)	1.023	1.003–1.043	0.022

*All models are adjusted for age, gender, migration status, subjective social status, education, general health literacy, and contact with COVID-19.

### COV-19-HL and Assessments of the effectiveness of protective measures against COVID-19

Participants rating the measures as rather effective or very effective had higher mean COV-19-HL scores than participants rating the measures as rather weak or not at all effective (see Table 6). The differences shown in bivariate analyses were significant in every item.

**Table 6:** COV-19-HL by assessment of measures

	Variables	COV-19-HL scores										ANOVA
		Mean (SD) and p (Scheffé Post-hoc-test)										*p*-value
		not at all		rather weak		neutral		rather strong		very strong		
Government requirements and recommendations	Closing of restaurants, stores, and businesses	34.03 (7.74)	ref.	36.61 (5.91)	<0.05	35.30 (5.65)	0.51	37.02 (5.40)	<0.01	39.21 (5.84)	<0.01	<0.01
	Ban on (public) events (sports, culture)	33.72 (8.40)	ref.	34.57 (5.96)	0.96	34.71 (5.70)	0.87	36.31 (5.47)	<0.05	38.90 (5.72)	<0.01	<0.01
	Restriction of social contacts	33.36 (8.00)	ref.	35.41 (5.09)	0.19	35.25 (5.60)	0.14	36.85 (5.52)	<0.01	39.67 (5.67)	<0.01	<0.01
	Thorough hand washing	30.06 (10.05)	ref.	35.44 (5.43)	0.06	32.97 (5.48)	0.46	35.38 (5.52)	<0.01	38.73 (5.71)	<0.01	<0.01
	Wearing oronasal masks	35.34 (7.27)	ref.	36.57 (5.79)	0.45	35.76 (5.60)	0.97	37.66 (5.48)	<0.01	39.10 (6.10)	<0.01	<0.01
	Physical distancing (1.5 meters min.)	32.86 (9.30)	ref.	35.11 (6.31)	0.54	33.53 (6.13)	0.99	36.25 (5.41)	<0.05	39.17 (5.60)	<0.01	<0.01
Additional measures	Surface disinfection (e.g. shopping carts, tables)	33.52 (8.50)	ref.	36.22 (6.27)	0.10	35.77 (5.93)	0.15	37.15 (5.33)	<0.01	39.42 (5.95)	<0.01	<0.01
	Wearing protective gloves	36.22 (7.37)	ref.	36.92 (5.33)	0.81	36.90 (5.91)	0.78	37.33 (5.42)	0.38	39.07 (6.31)	<0.01	<0.01
	Hand disinfection	33.77 (9.20)	ref.	36.20 (5.30)	0.47	35.66 (5.69)	0.59	36.87 (5.77)	0.09	38.64 (5.99)	<0.01	<0.01

As pointed out in the logistic regression models (see Table 7), higher COV-19-HL leads to an increased chance of rating a protective measure as effective. This is shown in the regression models of all items being adjusted for age, gender, migration status, subjective social status, education, general health literacy, and exposure to COVID-19. For example, with every additional score in COV-19-HL, the chance of rating thorough hand washing as effective is 1.107-times higher (CI 1.074–1.142, *p* < 0.01).

**Table 7:** Distribution of the assessments of protective measures and multivariate regression models exploring the assessments by COV-19-HL

				Regression models with COV-19-HL as independent variable*		
Assessment of the effectiveness of measures		No to neutral consent n (%)	Rather strong or very strong consent n (%)	OR	95% confidence interval	p
Government requirements and recommendations	Closing of restaurants, stores, and businesses	526 (33.5)	1044 (66.5)	1.083	1.061–1.106	<0.01
	Ban on (public) events (sports, culture)	318 (20.3)	1252 (79.7)	1.103	1.077–1.130	<0.01
	Restriction of social contacts	461 (29.4)	1109 (70.6)	1.096	1.072–1.120	<0.01
	Thorough hand washing	145 (9.2)	1425 (90.8)	1.107	1.074–1.142	<0.01
	Wearing oronasal masks	675 (43.0)	895 (57.0)	1.073	1.052–1.094	<0.01
	Physical distancing (1.5 meters min.)	247 (15.7)	1323 (84.3)	1.105	1.076–1.133	<0.01
Additional measures	Surface disinfection (e.g. shopping carts, tables)	530 (33.8)	1040 (66.2)	1.072	1.051–1.094	<0.01
	Wearing protective gloves	965 (61.5)	605 (38.5)	1.035	1.015–1.056	<0.01
	Hand disinfection	380 (24.2)	1190 (75.8)	1.057	1.035–1.080	<0.01

*All models are adjusted for age, gender, migration status, subjective social status, education, general health literacy, and contact with COVID-19.

## Discussion

### Discussion of the results

The results of this survey show that COV-19-HL can be gauged better than the general health literacy in Germany before the COVID-19 pandemic as assessed by previous studies (Schaeffer et al. 2016; Hurrelmann et al. 2020). This could be due to the omnipresent information in the media. In the HLS-GER study on health literacy in Germany, older persons were classified as a risk group with rather limited health literacy (Schaeffer et al. 2016; Hurrelmann et al. 2020). This finding could not be confirmed in our survey about COV-19-HL. However, we found small but significant differences in COV-19-HL by gender, migration status, education, and subjective social status. Another German survey on COV-19-HL found no significant differences in average COV-19-HL for the categories age, gender, education, household income, children under 18 in the household, and region of residence (Okan et al. 2020a). As mentioned earlier, there are possibly no major differences because of the ubiquitous media coverage on many platforms. Another reason as to why social differences in COV-19-HL are smaller than in the pre-pandemic overall health literacy might be that the recommendations for dealing with the pandemic are easy to understand and conduct.

In fact, the presence of chronic illness was associated with better COV-19-HL. This could be related to the fact that those with pre-existing diseases or who are aged 50–60 years and older are considered as a risk group for COVID-19 (Robert Koch Institut 2020c) and, therefore, are even more likely to inform themselves and exchange information. The authors of the HLS-GER survey present different results; they describe poor health literacy levels for people with chronic diseases still existing during the pandemic on a similar level as before the COVID-19 outbreak (Schaeffer et al. 2021). This inconsistency could possibly be explained by certain differences in the study sample, such as the fact that all the participants in our study are Internet users who might have a higher affinity to news.

Of the five knowledge questions posed in this survey, only 19% of the respondents were able to answer all of the questions correctly. With a look at the education levels, we also found greater inequality in knowledge about COVID-19 between educational groups. The participants faced difficulties particularly in rating the science-related statements where basic epidemiological knowledge is needed. There still seems to be a lack of knowledge about current developments in 2022. Only about a half of the participants in the COSMO survey (Universität Erfurt et al. 2022) were able to identify false statements about the Omicron variant. At the same time, self-perceived knowledge has already been at a high level in 2020 and still is according to this survey (Universität Erfurt et al. 2022).

Interestingly, the positive self-assessment of COV-19-HL only correlates weakly with the actual knowledge of COVID-19. Our results indicate that information was absorbed frequently and was perceived as adequate, so COVID-19-HL could be overestimated by the participants. Yet, there could be a lack of critical analysis and in-depth understanding of information. Our findings on the different levels of health literacy (see Fig. 1), as characterized by Nutbeam (2000), show that skills of critical literacy are less pronounced than skills of basic and communicative literacy. In this context, Abel and McQueen (2021) suggest a new definition of critical health literacy during pandemics. It comprises not only the competence to check if sources are trustworthy, but also the ability to make decisions in acute situations, and to adjust the pandemic recommendations to their own lives. Empirical studies are needed to explore how knowledge about a pandemic and (critical) health literacy are related.

The measures against COVID-19 such as physical distancing and closing of restaurants, stores, etc., during the first wave were overall estimated effective by the participants. The rather limited consent to oronasal masks could have been a result of the ongoing discussion about their necessity and effectiveness in reducing the spread of the virus in April 2020. According to our survey, a higher COV-19-HL correlates with a higher approval of the effectiveness of measures. This finding was similarly seen in other studies: People with higher health literacy seemed to show more positive attitudes toward preventive practices in the first months of the pandemic (McCaffery et al. 2020; Silva and Santos 2021). Furthermore, a higher health literacy was associated with increased adherence to preventive behaviors (Hermans et al. 2021; Patil et al. 2021). In summary, pandemic-related health literacy seems to encourage preventive attitudes and behaviors against a virus.

### Limitations

There are some limitations to consider with regard to the results from this survey on COV-19-HL. One main reason for limitations is that data was obtained by an online panel. There could be a risk of self-selection bias because all participants of the survey were panel members. Also, all of the respondents should be familiar with the possibilities of the Internet and searching for information online. People with no access to the Internet and very infrequent users were not part of the sample. These are predominantly older people. A survey in 2018 showed that only 55% of those from 70 years of age and older and 21% of those from 60 to 69 years of age do not use the Internet in Germany (Initiative D21 e.V. 2019). Therefore, the study sample represents German internet users (being panel members) and excludes those who are not online. Furthermore, in this sample, the proportion of people with low education is larger than the national average. It remains unclear if our conclusions on COV-19-HL are transferable to the overall population. Nevertheless, the sample is representative of the German population regarding age, gender, and residence in a federal state (not crossed) according to Eurostat 2018 database.

COV-19-HL was surveyed via self-assessment by the participants, so it is possible that the competencies were over- or underestimated. This becomes clear with regard to the evaluation of the knowledge questions, in which the participants did not perform as well as their calculated COV-19-HL would suggest. To date, there is also no gold standard of an instrument existing to survey pandemic-related health literacy. However, adoptions of the common health literacy scales are necessary to include specific topics such as the handling of huge amounts of information and fake information.

## Conclusion

The findings of this study contribute to the insights into social disparities in health literacy and to the understanding of correlations with pandemic-related knowledge and attitudes. Those with low education, low subjective social status, and migrants were more likely to have lower COV-19-HL and knowledge about COVID-19. Moreover, a potential benefit of pandemic-related health literacy was shown in that better COV-19-HL was associated with better knowledge and rating measures as effective. It was evident that a deeper understanding of COVID-19 and its prevention in the German population is vital, especially in disadvantaged groups. For this purpose, it is necessary to identify effective forms of health communication, i.e., which information channels, formats, and content are suitable for which target groups to increase health literacy. With regard to the results of the knowledge of COVID-19 and the assessment of measures in this study, further research should determine in greater depth what specific difficulties there are in understanding and evaluating information about a pandemic. This may identify factors that can be used in health communication to achieve equal opportunities in health literacy. Good health literacy across the population could be key to combating a pandemic.

## Data Availability

The datasets used and/or analyzed during the current study are available from the corresponding author on reasonable request.
